# Evaluation of how facial sunscreens are applied by the population: an approach beyond the product quantity^☆^

**DOI:** 10.1016/j.abd.2024.04.009

**Published:** 2024-11-15

**Authors:** Lucivaldo Fernandes Cruz, Catarina Silva Guimarães, Bianca Lemos Oliveira, Bruna Santana Neves, Caio Ferraz Cabral de Araújo, Maria Clara Botelho de Sousa, Vinicius Rosenbergre dos Santos Carmo, Juliano Geraldo Amaral, Gabriel Azevedo de Brito Damasceno

**Affiliations:** a Instituto Multidisciplinar em Saúde, Campus Anísio Teixeira, Universidade Federal da Bahia, Vitória da Conquista, BA, Brazil; b Programa de Pós-Graduação em Biociências, Campus Anísio Teixeira, Universidade Federal da Bahia, Vitória da Conquista, BA, Brazil

*Dear Editor,*

Regular use of sunscreen minimizes the harmful effects of ultraviolet radiation. The lack of sufficient sun protection means that the individual is vulnerable to the effects of solar radiation, which include both aesthetic and health-related problems.

Sunscreens are subjected to *in vivo* SPF which generally uses a standard of 2 mg/cm^2^ of skin.^1^ To achieve this protection, it is necessary to apply the correct amount in a uniform manner. When applied in insufficient quantities, the real SPF value is lower, which can lead to a false sensation of protection.

Therefore, the objective of this work was to evaluate, quantitatively, the quality of the method of applying facial photoprotectors and relate it to aspects of the studied population as a way of improving their education in this matter.

The study was carried out between November and December 2023 in Vitória da Conquista/BA (Ethics and Research Committee of the IMS-UFBA approval nº 69091123.7.0000.5556). The volunteers were instructed to apply a commercial sunscreen with SPF 70 in the amount and in the way they usually do. Then, a UV photograph of their face was taken by researchers using a special device that combines a UV camera with an integrated UVA light, providing sufficient illumination for standardized sunscreen coverage (UV LOOK, Youcai Technology Co). Finally, a study on sun habits and photoprotection care was carried out. The total facial surface and the surface where the sunscreen was applied were determined, in pixels, using the GIMP software – GNU Image Manipulation Program.

The surface area of sunscreen coverage was calculated by the quotient of the region where the sunscreen was applied to the total surface area of the volunteer's face, excluding the eye and lip regions (Eq. (1)). Statistical analysis was performed using GraphPad Prism 5.

(1) $$\text{Covering Surface}=\left(\frac{\text{Sunscreen coverage area}}{\text{Total face area}-\left(\text{Lip area+eye area}\right)}\right)\times 100\%$$

The present study was carried out on 177 volunteers (18 to 64 years old), 115 women (64.97%) and 62 men (35.03%), with an average age of 26.52 ± 8.96 years and the average application surface was calculated at 88.21 ± 23.83%.

Chemical filters absorb UV radiation and when using a device that filters visible light and captures UV radiation, the regions covered with sunscreen appear darker.^2^ Fig. 1 shows an example of how the area of application of the sunscreen was visualized, in the form of an increasing scale.

**Fig. 1:**
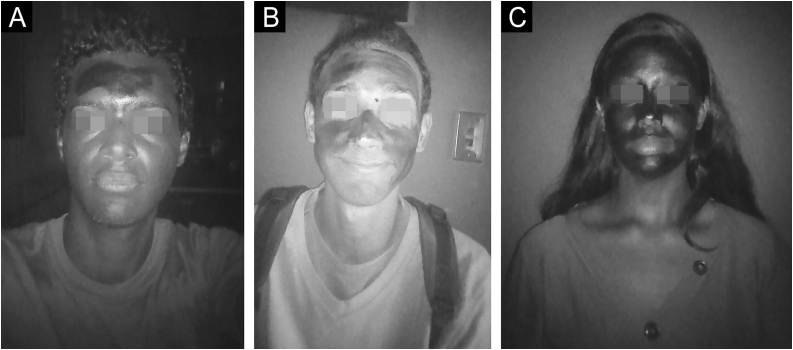
Increasing scale of area of application of the sunscreen. *Areas shaded in black on the face of the volunteer corresponding to (A) 15.53%, (B) 56.19% and (C) 100% of sunscreen surface cover.

Regarding the analysis of the results based on the answers obtained from the questionnaires, it was possible to observe certain scenarios. Women presented an average application surface of 92.90 ± 15.59%, while men reached values of 79.50 ± 32.65%, these values were statistically significant (unpaired *t*-test and p-value < 0.05). Similar results were observed by Jovanovic, Schornstein,^3^ when evaluating total body coverage, in that women had significantly greater coverage than men. Women are known for taking greater care of their health compared to men.^4^ It is predicted that by the year 2040 men will present 26% more cases of melanoma with 36% more mortality when compared to women.^5^ Additionally, a study carried out with 705 men found that 83% of them did not use sunscreen daily and only 38% reported using the product weekly.^6^ This factor of taking less precautions on the part of men was also observed in the present study.

Another important aspect is the age range of the volunteers. Due to the large difference in volunteers in each group, it was not possible to carry out statistical analysis using the age stratification shown in Fig. 2. However, even without presenting statistically significant variations, the best results were obtained by the 35 to 46-year-old group, obtaining up to 94% of surface coverage.

**Fig. 2:**
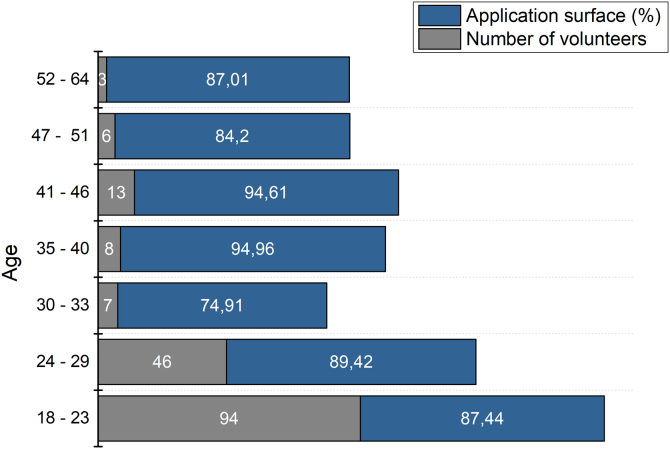
Application surface depending on the age of volunteers.

A study carried out with 5992 volunteers used questionnaires on skin cancer and preventive measures and showed that older people and those with less access to education, or with a non-formal education, assume that they were exposed to lower risks for skin cancer.^4^ However, this was not reflected in the results of the surface area sunscreen application of our study.

Finally, volunteers were classified according to their self-declared skin color (Fig. 3). It is possible to observe a greater surface area of sunscreen coverage in people with white and brown skin. However, this difference was not statistically significant (Anova + Tukey).

**Fig. 3:**
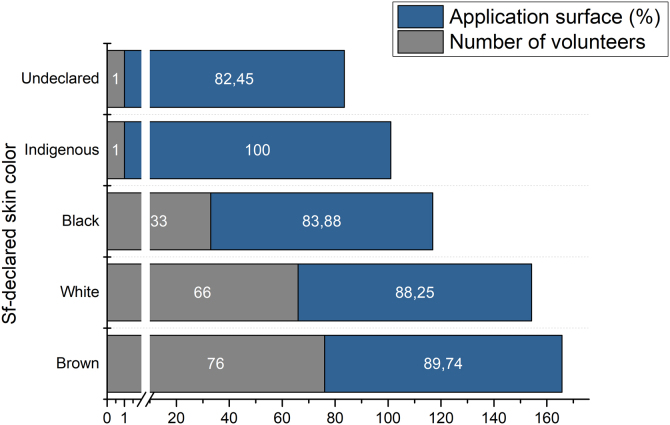
Application surface depending on the skin color of the volunteers.

People with lighter skin are more likely to burn and more sensitive to UV radiation, while this is less likely to happen to people with darker skin, according to the skin phototype classification established by Thomas B. Fizpartrick.^7^ Thus, it is common to observe that people with dark skin tend to protect themselves less against solar radiation due to the characteristics of this skin phenotype, which was observed for people with black skin.

Just as important as the application is the reapplication. Thus, we also investigated the frequency of sunscreen application by volunteers, the frequency of reapplication, and the average duration of a bottle of sunscreen, as a way of estimating the amount applied and photoprotection care habits (Table 1).^2^, ^8^

**Table 1:** Frequency of application of sunscreens.

Classification	Number of Volunteers	Percentage in population	Covering surface
Daily	60	33.90%	90.56 ± 22.57%
When exposed to the sun	65	36.72%	83.60 ± 22.62%
Sporadically	24	13.56%	88.40 ± 21.81%
Undefined	28	15.82%	86.30 ± 3.37%

It was observed that even those people who use sunscreen irregularly achieved high levels of product coverage but those who use sunscreens daily achieved greater application coverage. However, when asked about the frequency of reapplication of the product, 92.66% (164 people) of volunteers stated that they do not have the habit of reapplying sunscreen.

According to the Brazilian Consensus on Photoprotection, the amount of 2 mg/cm^2^ for *in vivo* tests is used because it generates a layer 1 millimeter thick across the entire facial epidermis. Therefore, we can estimate that the correct use of sunscreen daily on the face would consume a 40 g bottle of product in approximately 30 days. In the present study, most volunteers exceed this period (Table 2), lasting up to one year, thus suggesting the application of the product in quantities well below the recommended amount.

**Table 2:** Average duration of facial sunscreens in real conditions of use by volunteers.

Estimated duration	Number of volunteers	Percentage
1 to 3 weeks	7	3.95%
1 to 3 months	104	58.75%
4 to 12 months	45	25.42%
Undefined	21	11.86%

In this work, although products up to the value of SPF 90 were cited, the range of SPF 30 and 50 are the most used by 84.75% of volunteers, being close to the minimum recommended by the Brazilian Society of Dermatology. There must be a broad-spectrum factor for UVA and UVB rays with an SPF at least equal to or greater than 30.^9^

It is possible to conclude that the evaluated population is sufficiently educated regarding the correct way to apply sunscreen, given the high percentage of surface area covered by photoprotector application. Among the parameters of gender, age, and skin color, only gender was significant, with women having a greater surface area for applying sunscreen. However, the average duration of the products and the absence of reapplication are still aspects that directly affect the quality of protection over the period that exposure occurs. This demonstrates that knowing how to apply sunscreen is not enough for effective protection. Furthermore, it was possible to demonstrate the applicability of a cheap and easy-to-use device for monitoring the surface of sunscreen application. These can be used by healthcare professionals at home since it has already been demonstrated that the use of UV photography devices improves photoprotective skin care.^10^ It is essential that scientific information reaches the population and these data can encourage educational campaigns about the correct use of photoprotectors at different levels of society.

## Financial support

None declared.

## Authors’ contributions

Lucivaldo Fernandes Cruz: Data collection, or analysis and interpretation of data; writing of the manuscript or critical review of important intellectual content; critical review of the literature.

Catarina Silva Guimarães: Data collection, or analysis and interpretation of data; writing of the manuscript or critical review of important intellectual content; critical review of the literature.

Bianca Lemos Oliveira: Data collection, or analysis and interpretation of data; writing of the manuscript or critical review of important intellectual content; critical review of the literature.

Bruna Santana Neves: Data collection, or analysis and interpretation of data; writing of the manuscript or critical review of important intellectual content; critical review of the literature.

Caio Ferraz Cabral de Araújo: Data collection, or analysis and interpretation of data; writing of the manuscript or critical review of important intellectual content; critical review of the literature.

Maria Clara Botelho de Sousa: Data collection, or analysis and interpretation of data; writing of the manuscript or critical review of important intellectual content; critical review of the literature.

Vinicius Rosenbergre dos Santos Carmo: Data collection, or analysis and interpretation of data; writing of the manuscript or critical review of important intellectual content; critical review of the literature.

Juliano Geraldo Amaral: Effective participation in the research guidance; final approval of the final version of the manuscript.

Gabriel Azevedo de Brito Damasceno: The study concept and design; statistical analysis; writing of the manuscript or critical review of important intellectual content; effective participation in the research guidance; final approval of the final version of the manuscript.

## Conflicts of interest

None declared.
